# Maternal dietary diversity and pattern during pregnancy is associated with low infant birth weight in the Cape Coast metropolitan hospital, Ghana: A hospital based cross-sectional study

**DOI:** 10.1016/j.heliyon.2020.e03923

**Published:** 2020-05-08

**Authors:** Dan Yedu Quansah, Daniel Boateng

**Affiliations:** a Department of Biomedical Sciences, School of Allied Health Sciences, University of Cape Coast, Cape Coast, Ghana; b School of Public Health, Kwame Nkrumah University of Science and Technology, Kumasi, Ghana

**Keywords:** Nutrition, Diet, Public health, Epidemiology, Dietary diversity score, Dietary patterns, Postnatal care, Low birth weight, Cape Coast

## Abstract

**Background:**

This study investigated the associations between mother's dietary diversity score and dietary patterns during pregnancy and the odds of low birth weight at the Cape Coast Metropolitan Hospital in Ghana.

**Methods:**

Mothers attending the postnatal clinic from January to August 2016 at the Cape Coast Metropolitan Hospital were included. Dietary information during pregnancy was assessed with a food frequency questionnaire. In reference to the women's dietary diversity score, women were categorized into low, medium or high dietary diversity score groups. The primary outcome was low birth weight and was defined as weight <2500 g at birth. Factor analysis was conducted to identify maternal dietary patterns and a multivariable logistic regression analysis was used to determine the associations between dietary diversity score and dietary patterns with low birth weight.

**Results:**

The prevalence of low birth weight in infants was 43.8% (95% CI = 39%–49%). After adjusting for covariates, the odds of low birth weight was four times higher in the low dietary diversity score group compared to the high dietary diversity score group (odds ratio [OR] = 4.29, 95% confidence interval [CI], 1.24–6.48). Three dietary patterns namely "Western", "Traditional" and "Healthy", which explained 58.23% of the total variance in food intake were identified. The subjects in the highest quartiles of “healthy” and “traditional” dietary pattern scores had significantly lower odds of low birth weight (healthy: OR = 0.23, 95% CI, 0.19–0.39, *P* trend <0.0001; traditional: OR = 0.14, 95% CI, 0.06–0.35, *P* trend <0.0001, respectively) compared to those in the lowest quartiles of dietary pattern score.

**Conclusion:**

Low dietary diversity score during pregnancy was associated with higher odds of infant low birth weight whereas dietary patterns considered as “healthy” and “traditional” during pregnancy were associated with lower odds of infant low birth weight. Findings of this study suggests that higher dietary diversity and “healthy” and “traditional” dietary patterns during pregnancy may be protective of LBW in the study area.

## 1 Introduction

The World Health Organization (WHO) defines low birth weight (LBW) as weight at birth less than 2500 g (5.5 pounds) (WHO, 1992). Research based on the theory of Developmental Origins of Health and Diseases (DOHaD) suggests that infants with LBW have a higher risk of developing hypertension, obesity, diabetes and mortality in adult life compared to infants with normal birth weight (Jornayvaz et al., 2016; Kopec et al., 2017; Tian et al., 2006). Factors such as socio-economic characteristics, mothers’ nutritional status before and during pregnancy and maternal anthropometric characteristics have been reported as risk factors of LBW (da Silva Lopes et al., 2017; Amegah et al., 2017). Interactions between maternal socio-economic characteristics and nutritional status during pregnancy are also known to influence LBW (Valero de Bernabé a et al., 2004). According to the WHO, poor maternal nutritional status during pregnancy confers greater risk for LBW in developing countries including Ghana (WHO, 2004).

Adequate food or nutrients intake during pregnancy provides maternal nutrient reserves that can serve as nutritional stores during pregnancy (Fowles and Gabrielson, 2005; Kant, 2004). Even though it is useful to isolate the impact of specific nutrients or foods, the results might be insufficient (Binkley et al., 2009; Farrell et al., 2009) to account for the complex behavior of food consumption and nutrient interactions during pregnancy (Loy and Mohamed, 2013). Therefore, dietary diversity and dietary patterns rather than single nutrient or food group might be useful to understand the relationship between overall maternal nutrition adequacy during pregnancy and birth outcomes especially in regions where diet assessments are complex and often times difficult (Hu, 2002).

Dietary diversity score (DDS) is a useful indicator of nutrient adequacy or overall diet quality (Ruel, 2002). Consuming diverse diet from different food groups and sources during pregnancy is an important approach to improve dietary quality and micronutrient status during pregnancy (FAO & FHI, 2016; Christian and Stewart, 2010). In a recent prospective cohort study involving women in Ethiopia, the attainment of ≥ four (4) DDS during pregnancy was inversely associated with the risk of maternal anemia, LBW, and pre-term birth. On the other hand, women with lower DDS during pregnancy had higher odds of delivering higher proportion of infants with LBW compared to those who had medium and higher DDS (Rammohan et al., 2017).

The use of dietary pattern analysis to understand nutritional intake and maternal health outcomes is becoming more popular in recent times as a way of deconstructing the complexity of diet and its relation to health. Derived using analytic methods such as principal component analysis (PCA), dietary patterns present a broader picture of food and nutrient consumption and is more predictive of disease risk than individual foods or nutrients (Hu, 2002; Cespedes and Hu, 2015; Zhang et al., 2018). Dietary patterns that are considered as “nutrient dense, “protein rich”, “health conscious”, and “Mediterranean” are found to be inversely associated with LBW (Chen et al., 2016a, Chen et al., 2016b). A recent review of literature also revealed that dietary patterns that consist of higher intakes of fruits, vegetables, legumes and fish had positive pregnancy outcomes including normal birth weight (Kjøllesdal and Holmboe-Ottesen, 2014).

In Ghana, some studies have investigated the relationship between socio-economic characteristics (Afriyie et al., 2016; Prah et al., 2016), maternal risk factors (Fosu et al., 2013), the effects of single nutrients (Amegah et al., 2017) and food groups during pregnancy with LBW. However, data on the relationship between DDS and dietary patterns during pregnancy have received little attention (Saaka, 2012; Abubakari and Jahn, 2016). This study therefore investigated the associations of maternal DDS and dietary patterns during pregnancy with LBW in the Cape Coast Metropolitan Hospital, a tertiary (referral) hospital in the Cape Coast metropolis of Ghana.

## 2 Methods

### 2.1 Study setting

We conducted this study in the Cape Coast metropolitan hospital in Ghana. Cape Coast is the capital of the central region located in southern Ghana. Health Services in the metropolis are provided by both government and private institutions and are structured along the three-tier system of the Primary Health Care strategy.

### 2.2 Study design and participants selection

This was a hospital based cross-sectional study. Our study population were mothers attending postnatal clinic at the Cape Coast metropolitan hospital during the study period (from January to August 2016). We included mothers ≥17 years who were attending postnatal clinic after they had attended Antenatal clinic (ANC) and delivered at the same facility. We systematically sampled eligible participants based on the midwife's appointment register on postnatal clinic days. Out of the total women who attended ANC and delivered at the facility, 483 mothers agreed to participate in the study. We excluded mothers who had stillbirths, delivered babies with gross congenital malformations and those who delivered infants with higher birth weight. Overall, 420 mothers were included in the final analysis.

### 2.3 Outcome variable

The main outcome of the study was infant birth weight. We obtained newborn birth weight and other data including neonatal sex from mothers’ birth records. The hospital delivery team recorded this information within 24 h after delivery. We dichotomized infant birth weight as low (<2500g) and normal (WHO, 1992).

### 2.4 Data collection

We administered a structured pretested questionnaire to elicit information on socio-demographic and lifestyle characteristics from the participants after explaining to them the objective of the study. A trained interviewer administered the questionnaire to mothers who could not read or write whereas questionnaires were self-administered by women who could read and write. We conducted all interviews in the office of the midwife after mothers’ postnatal appointment. The Research Committee of the Department of Human Biology, University of Cape Coast approved the study protocol and we obtained signed informed consents from all participants.

### 2.5 Anthropometric measurements

We obtained maternal height and weight during pregnancy from mothers’ ANC records. Height was measured during the first ANC appointment with a stadiometer and the value was rounded to the nearest 0.1 cm whereas weight was measured at the first ANC appointment and at the last ANC prior to delivery using a Tanita HD-351 scale that was regularly calibrated and was rounded to the nearest 0.05 kg. Self-reported pre-pregnancy weight highly correlated with weight measured during pregnancy. Weight gain during pregnancy was determined by subtracting weight at first ANC from the weight at last ANC prior to delivery. Pre-pregnancy body mass index (BMI) was categorized into underweight (<18.50 kg/m^2^), normal (18.50–24.99 kg/m^2^), overweight (25.00–29.99 kg/m^2^) and obese (≥30 kg/m^2^) (WHO, 2000). The number of ANC visits during pregnancy was categorized into 1–4 times, 5–8 times and >8 times.

### 2.6 Measurement of covariates

Information on mother's demographic characteristics including education, income and marital status were obtained with a questionnaire. Educational level was categorized into no formal education, primary and/or junior high school (JHS) (grade 1–9), senior high school (SHS) (grade 10–12) and tertiary education (diploma or university degree and above). Monthly household income was self-reported by study participants, and was grouped into three categories: ≤ GH¢ 300.00, GH¢ 301.00–500.00 and GH¢ more than 500.00. The lowest income level (GH¢ 300.00) was twice the national minimum wage of GH¢ 162.00 ($1 = GH¢ 4.84) at the time of the study. We categorized information on marital status into “married or single”. Data on birth-order (1, 2, 3 and above), parity (1, 2, 3 and above), smoking (“yes” or “no”), alcohol intake (“yes” or “no”), and supplement intake during pregnancy (“yes” or “no”) were also extracted from patients medical chats and records.

### 2.7 Maternal dietary diversity scores and dietary patterns

We assessed dietary information during pregnancy with a food frequency questionnaire (FFQ). The FFQ contained 52 food items including cereals, milk and dairy products, fats and oils, legumes, tubers, meats, eggs, fruits, fish and vegetables. We adjusted these food items to accommodate the most common foods eaten among the people of the metropolis in consultation with the metropolis nutritionist. We excluded beverages such as coffee, tea and sweets as the study population did not commonly consume them. Frequency responses were ‘≥2 times/day, ‘once/day’, ‘2–4 times/week’, ‘occasionally’ and ‘not at all’.

To determine maternal DDS, we made use of the Minimum Dietary Diversity for women of reproductive age (MDD-W). We aggregated the food items on the FFQ into 10 food groups as outlined by the Food and Agriculture Organization (FAO) (FAO & FHI, 2016). The 10 resulting food groups were grains (bread, maize, sorghum, and rice), beans (all kinds of beans, other legume products), dairy products (powdered and liquid forms of milk, cheese, and yoghurt), meat (poultry products, fish, pork, chicken, goat, and guinea fowl), egg (chicken, duck, guinea fowl). Green leafy vegetables (broccoli, *kontomire*, carrot greens, chili, lettuce, okra), other vegetables (mushroom, onion, tomato, peas, green pepper, eggplant, and cabbage), fruits rich in vitamin A (pumpkin, pawpaw, mango, apricot, melon, peaches, and tomato), other fruits (apple, banana, avocado, coconut flesh, guava, orange, pear, watermelon, tangerine) and nuts and seeds (groundnut or peanuts, almond, cashew, sesame seed).

Each food group was assigned a score of 1 if a subject had consumed at least one of the food items in that particular food group or 0 if not. Based on this algorithm mothers were classified into a low, medium or high DDS group if they consumed 5 or less food groups (DDS ≤5), between 6 to 8 food groups (DDS = 6–8), and 8 or more food groups (DDS ≥8), respectively.

We identified three dietary patterns that best explained the dietary intake of participants during pregnancy; these were "Western", "traditional" and "healthy" patterns based on the food items that correlated with the factors. The "Western" dietary pattern was characterized by high intakes of meat, poultry products, organ meat (liver, kidney), egg and pork, whereas the "Traditional" dietary pattern was highly correlated with high intakes of maize, fish, shell fish, sorghum, rice, bread, milk and other vegetables. The "Healthy" dietary pattern had high correlation with green leafy vegetables, fruits rich in vitamin A, pumpkins and other fruits.

### 2.8 Statistical analysis

We performed all statistical analyses with SPSS software version 22 (IBM Corp, 2013). The prevalence of LBW according to socio-demographic and health characteristics were described using frequency and percentages. Dependent and independent variables were normally distributed. Pre-pregnancy weight and weight measured during ANC were highly correlated (r = 0.9, p < 0.001). The differences in socio-demographic and health characteristics according to DDS and neonatal birth weight were determined using Pearson Chi-square test. We identified maternal dietary patterns by performing a factor analysis using PCA. Varimax rotation was used and the distributions of scores were centered on 0 with standard deviation of 1 for easy interpretation. We observed no intersection of loading across dietary patterns. Factor loadings had magnitude >0.3. The percentage of variance explained for "Western", "traditional" and "healthy" patterns were 19.78%, 12.65% and 12.30% respectively with accumulated variability of 58.23%.

We performed a logistic regression analysis to determine the association between LBW and DDS and dietary patterns. Socio-demographic and lifestyle characteristics were included in the multivariable analysis as potential confounders. In model 1, we adjusted for age, education, employment, marital status, and monthly income. In model 2, we adjusted for covariates in model 1 as well as birth-order, parity and ANC attendance whereas in model 3 we further adjusted for smoking, alcohol intake and supplement intake. We calculated p-value for linear trend based on the median value of the regression scores of each dietary pattern and statistical significance was accepted at P < 0.05.

## 3 Results

### 3.1 Characteristics of study participants by birth outcome

This study had a response rate of 85% (i.e. 483 out of 568 women agreed to participate). Table 1 shows the baseline characteristics of subjects. The mean age (±standard deviation) of subjects was 26.7 ± 5.7 years with a range of 17–45 years. Majority (37.4%) had primary school education followed by tertiary education (30.5%). Most of the subjects were employed (68.8%), married (66.2%) and had low monthly income (51.2%). Majority (75.2%) were overweight before pregnancy (25.0–29.99 kg/m^2^) and gained (weight) less than 12kg during pregnancy (76.4%). Mothers who were single (p < 0.0001), were unemployed (p = 0.0050) and those who gained <12kg during pregnancy (p = 0.0220) had significantly higher prevalence of LBW than those who were married, employed and gained ≥12kg during pregnancy respectively.

**Table 1:** General characteristics of study participants according to neonatal birth weight.

Variables	Frequency (N)	Percent (%)	Birth weight		*P*-value
			Low (n = 184) (N, %)	Normal (n = 236) (N, %)	
Age, years					

<20	16	3.8	6 (37.5)	10 (62.5)	<0.0001
20-30	322	76.7	162 (50.3)	160 (49.7)	
>30	82	19.5	16 (19.5)	66 (80.5)	

Educational level∗					

No formal	68	16.2	41 (60.3)	27 (39.7)	<0.0001
Primary/JHS	157	37.4	69 (43.9)	88 (56.1)	
SHS	67	16.0	16 (23.9)	51 (76.1)	
Tertiary	128	30.5	58 (45.3)	70 (54.7)	

Employment					

Yes	289	68.8	114 (39.4)	175 (60.6)	0.0050
No	131	31.2	70 (53.4)	61 (46.6)	

Marital status					

Single	142	33.8	82 (57.7)	60 (42.3)	<0.0001
Married	278	66.2	102 (36.7)	176 (63.3)	

Monthly household income (GH¢)					

≤300	215	51.2	109 (50.7)	106 (49.3)	<0.0001
301-500	83	19.8	47 (56.6)	36 (43.4)	
500+	116	27.6	28 (24.1)	88 (75.9)	

Birth order					

1	236	56.2	124 (52.5)	112 (47.5)	<0.0001
2	141	33.6	47 (33.3)	94 (66.7)	
3+	43	10.2	13 (30.2)	30 (69.8)	

Parity					

1	239	57.9	124 (51.9)	115 (48.1)	<0.0001
2	106	25.2	25 (23.6)	81 (76.4)	
3+	75	17.9	35 (46.7)	40 (53.3)	

Antenatal clinic attendance (n = 408)					

1–4 times	24	5.9	3 (12.5)	21 (87.5)	0.095
5–8 times	245	60.0	36 (14.7)	209 (85.3)	
>8times	139	34.1	32 (23.0)	107 (77.0)	

Smoking during pregnancy					

Yes	32	7.6	10 (31.3)	22 (68.8)	0.028
No	388	92.4	61 (15.7)	327 (84.3)	

Alcohol intake during pregnancy					

Yes	44	10.7	12 (27.3)	32 (72.7)	0.028
No	368	89.3	53 (14.4)	315 (85.6)	

Pre pregnancy BMI (kg/m^2^)‡					

Underweight (<18.50)	10	2.4	12 (66.7)	6 (33.3)	0.0258
Normal (18.5–24.99)	59	14.1	85 (41.7)	119 (58.3)	
Overweight (25.0–29.9)	315	75.2	47 (39.5)	72 (60.5)	
Obese (≥30)	35	8.4	40 (50.6)	39 (49.4)	

Gestational weight gain (Kg)					

<12	321	76.4	125 (45.2)	176 (54.8)	0.0220
>12	89	21.2	29 (32.6)	60 (67.4)	

Neonatal Sex					

Male	306	72.9	166 (54.2)	140 (45.8)	<0.0001
Female	114	27.1	18 (15.8)	96 (84.2)	

P-value were derived from chi-square test.

∗ JHS meant subjects with educational level from grade 1–9 called the junior high school, SHS meant education level from grade 10–12 which is called the senior high school and Tertiary meant subject with a diploma or university degree and above.

‡ Pre-pregnancy BMI meant body mass index before pregnancy.

### 3.2 Association between DDS and LBW

**Figure 1:**
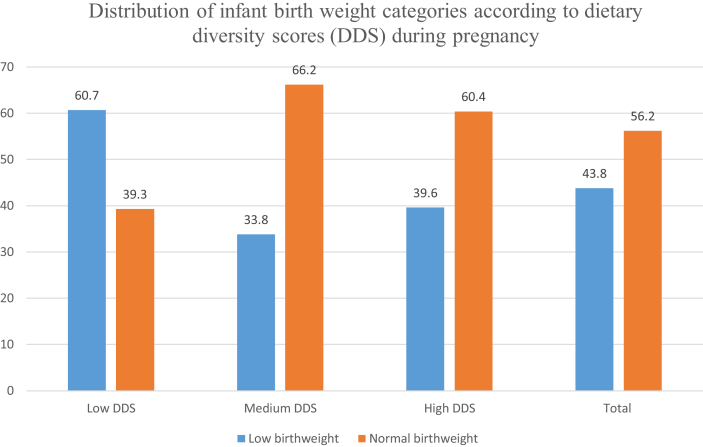
Figure 1 describes the distribution of infant birth weight categories (low vs. normal) according to maternal dietary diversity score (DDS) during pregnancy. The overall proportion of low birth weight (LBW) was 43.8%. The proportion of LBW was higher in women who had low DDS during pregnancy (60.7%) compared to women who had high DDS during pregnancy (39.6%) (p < 0.001).

Figure 1 describes the distribution of infant birth weight categories (low vs. normal) according to maternal DDS during pregnancy. The overall proportion of LBW was 43.8% (95% CI = 39%–49%). The proportion of LBW was higher in women who had low DDS during pregnancy (60.7%) compared to women who had high DDS during pregnancy (39.6%) (p < 0.001).

Table 2 shows the results of the odds ratios (OR) and 95% confidence intervals (CI) of LBW according to DDS. Mothers with lower DDS had significantly higher odds of delivering infants with LBW compared to those who had higher DDS in both the unadjusted (crude) model (OR: 2.35, 95% CI: 1.23–4.47) and in model 1 (OR: 1.99, 95%CI: 1.02–3.90) and model 2 (OR: 2.14, 95%CI: 1.07–4.27). In the fully adjusted model 3, the odds of LBW was higher in the low DDS group compared to those who had high DDS (OR = 4.29 95% CI, 1.24–6.48).

**Table 2:** Odds ratios (OR) and 95% confidence interval (CI) of LBW risk according to DDS (N = 420).

Dietary diversity score	OR (95%, CI)	P-value
Crude∗		

Low	2.35 (1.23–4.47)	0.009
Medium	0.77 (0.42–1.44)	0.424
High (referent)	1.00	

Model 1†		

Low	1.99 (1.02–3.90)	0.043
Medium	0.77 (0.41–1.47)	0.442
High (referent)	1.00	

Model 2‡		

Low	2.14 (1.07–4.27)	0.031
Medium	0.87 (0.45–1.69)	0.687
High (referent)	1.00	

Model 3|		
Low	4.29 (1.24–6.48)	0.021
Medium	0.92 (0.28–2.96)	0.892
High (referent)	1.00	

CI, confidence interval; OR, odds ratio; ref, referent.

∗ Crude meant univariate unadjusted regression estimates.

† Model 1: Adjusted for socio-demographic characteristics including age, education, employment, marital status and monthly income.

‡ Model 2: Model 1 + birth-order of infant, parity of infant and number of ante-natal clinic (ANC) attendance.

| Model 3: Model 2 + smoking, alcohol intake and supplement intake.

### 3.3 Association between dietary patterns during pregnancy and LBW

**Figure 2:**
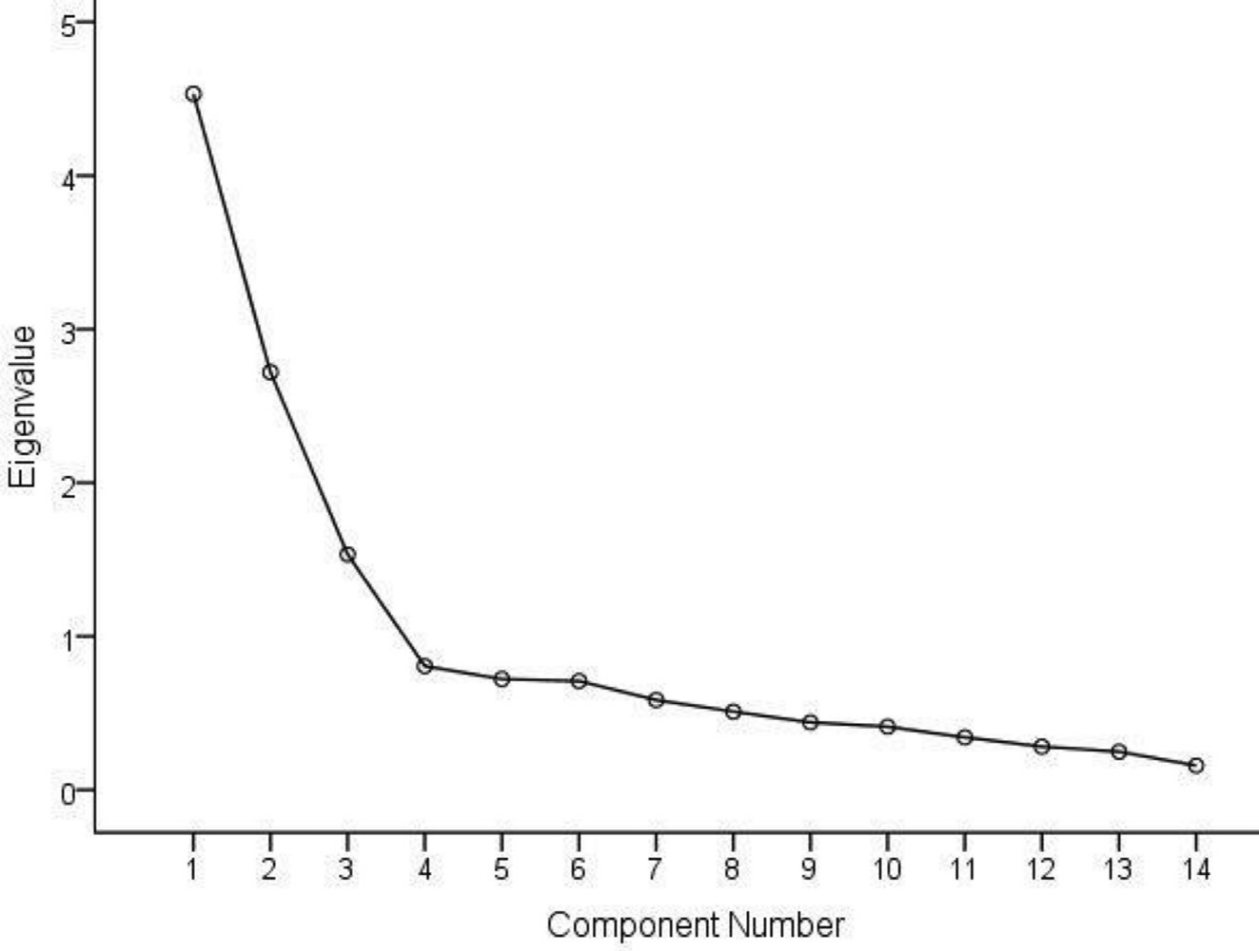
Scree plot showing the eigenvalues of the three main dietary patterns from the principal components analysis.

We identified three dietary patterns that best explained the dietary intake of participants during pregnancy in this study. Figure 2 is a scree plot showing the eigenvalues of the three main dietary patterns obtained from the principal components analysis. These three factors were "Western", "traditional" and "healthy" patterns. Table 3 shows the factor loading of each food item. Table 4 shows the ORs and 95% CI of LBW according to the quartiles (Q) of each dietary pattern score. In the unadjusted (crude) regression model, the highest quartile of dietary pattern score in both the “healthy” and “traditional” dietary patterns (healthy: OR = 0.33 95% CI, 0.32–0.91, P trend <0.0001; traditional: OR = 0.26 95% CI, 0.14–0.47, P trend <0.0001) were significantly associated with lower odds of LBW. These observed associations were consistent in the adjusted models. In model 3, the highest quartile of “healthy” and “traditional” dietary pattern scores were significantly associated with lower odds of LBW (healthy: OR = 0.23 95% CI, 0.19–0.39, P trend <0.0001; traditional: OR = 0.14 95% CI, 0.06–0.35, P trend <0.0001). In the “traditional” dietary pattern, the odds of LBW consistently decreased with the introduction of additional covariates in the regression model but remained significant. We found no significant association between the “Western” dietary pattern and the odds of LBW both crude and adjusted regression models.

**Table 3:** Loading factors from factor analysis using varimax rotation.

	Factor loading		
	Western	Traditional	Healthy or prudent
Meat	0.931		
Poultry products	0.927		
Organ meat (liver, kidney)	0.583		
Egg	0.711		
Pork	0.612		
Maize		0.621	
Shell fish		0.569	
Sorghum		0.518	
Rice		0.510	
Fish		0.453	
Bread		0.423	
Milk		0.321	
Other vegetables		0.311	
Green leaves			0.819
Fruits			0.675
Pumpkins			0.505
other fruits			0.486

Eigenvalues	4.51	2.72	1.53
% of variance explained	19.78	12.65	12.30

Kaiser-Meyer-Olkin Measure of Sampling Adequacy = 0.660, Bartlett's Test of Sphericity. Chi-Square = 2254.851, P = <0.0001.

**Table 4:** Odds ratio (OR) and 95% confidence interval (CI) of the odds of LBW according to the quartile (Q) of dietary pattern score.

Dietary pattern	Q1 (n = 105)	Q2 (n = 105)	Q3 (n = 105)	Q4 (n = 105)	*P* for trend
		OR (95%, CI)	OR (95%, CI)	OR (95%, CI)	
Healthy	(ref)				

Crude∗	1.00	0.64 (0.55–0.84)	0.47 (0.35–0.72)	0.33 (0.32–0.91)	<0.0001
Model 1†	1.00	0.60 (0.54–0.84)	0.57 (0.53–0.94)	0.45 (0.41–0.87)	<0.0001
Model 2‡	1.00	0.44 (0.32–0.92)	0.33 (0.31–0.62)	0.38 (0.23–0.72)	<0.0001
Model 3|	1.00	0.33 (0.21–0.59)	0.42 (0.36–0.84)	0.23 (0.19–0.39)	<0.0001

Traditional					

Crude	1.00	0.30 (0.17–0.56)	0.46 (0.26–0.81)	0.26 (0.14–0.47)	0.0020
Model 1	1.00	0.16 (0.07–0.36)	0.34 (0.16–0.70)	0.18 (0.08–0.40)	<0.0001
Model 2	1.00	0.14 (0.06–0.33)	0.31 (0.14–0.70)	0.15 (0.06–0.37)	<0.0001
Model 3	1.00	0.13 (0.05–0.31)	0.30 (0.13–0.68)	0.14 (0.06–0.35)	<0.0001

Western					

Crude	1.00	0.71 (0.39–1.29)	2.41 (1.36–4.26)	1.71 (0.97–3.00)	0.5310
Model 1	1.00	0.79 (0.35–1.76)	1.69 (0.73–3.88)	1.13 (0.51–2.48)	0.2270
Model 2	1.00	0.77 (0.32–1.86)	1.38 (0.57–3.32)	1.31 (0.55–3.08)	0.1560
Model 3	1.00	0.75 (0.30–1.84)	1.48 (0.60–3.60)	1.73 (0.68–4.37)	0.1580

CI, confidence interval; OR, odds ratio; ref, referent.

∗ Crude meant univariate unadjusted regression estimates.

† Model 1: Adjusted for socio-demographic characteristics including age, education, employment, marital status and monthly income.

‡ Model 2: Model 1 + birth-order of infant, parity of infant and number of antenatal care (ANC) attendance.

| Model 3: Model 2 + smoking, alcohol intake and supplement intake.

## 4 Discussion

This hospital based cross-sectional study investigated the association between maternal DDS and dietary patterns with LBW among postnatal women. To our knowledge, this is the first study to investigate the relationship between both DDS and dietary patterns with infant LBW simultaneously in a clinic setting. We found a protective relationship between maternal DDS and dietary patterns during pregnancy with the odds of LBW. The odds of LBW was higher in women who had low DDS and mothers who had “healthy” or “traditional” dietary patterns during pregnancy had lower odds of delivering an infant with LBW.

Previous studies have shown that the diversity of foods eaten during pregnancy influences birth outcomes including birth weight (Kolte et al., 2009; Andersen et al., 2003; Zerfu et al., 2016) and Apgar score (Quansah et al., 2019). High dietary diversity diet during pregnancy provides adequate micronutrients that are important for the growth and development of the fetus. In this study, mothers who had low DDS had higher odds of LBW than those who had high DDS scores. The consumption of fewer food groups could result in deficiencies for essential micronutrients that are important for the growth and development of the fetus. Consistently, findings of other studies as well as our results suggests that DDS could be an independent predictor of birth outcomes including neonatal birth weight in developing countries including Ghana (Saaka, 2012; Abubakari and Jahn, 2016; Ali et al., 2014).

We also found that sociodemographic characteristics including marital status, unemployment lower household income and no formal education were significantly associated with lower DDS. Non-educated women are more likely to consume less diverse diet compared to those who were educated. Reasons such as cultural taboos might influence the food choices of women with less education and could lead to less dietary diversity. A recent study has also showed that inadequate dietary diversity is significantly higher among women with lower income (Yeneabat et al., 2019).

In this study, mothers in the highest quartile of “healthy” and “traditional” dietary pattern scores showed lower odds of delivering an infant with LBW. The “healthy” and “traditional patterns” identified in this study consisted of foods that are similar to “traditional” and “healthy” dietary patterns identified in other epidemiologic studies (Kjøllesdal and Ottesen, 2014). The nutrient-dense nature of the “healthy” and “traditional” dietary patterns might contribute to the protective association observed for LBW in this study. This is consistent with a study in South Africa where a healthier dietary pattern was inversely associated with LBW (Annan et al., 2015). In a recent systematic review, dietary patterns that consisted of higher intakes of fruits, vegetables, legumes and fish led to positive pregnancy outcomes (Chen et al., 2016a, Chen et al., 2016b). Thompson and colleagues also reported that mothers who had higher “traditional” dietary pattern in early pregnancy were less likely to deliver newborns with LBW (Thompson et al., 2010) whereas a previous study indicated that both "Health conscious" and "non-Health conscious" dietary patterns offered protective effect for LBW among northern Ghanaian women (Abubakari and Jahn, 2016). We however did not find an association between the “Western” dietary pattern and LBW although the “Western” dietary pattern consisted of foods that were high in protein, zinc (for growth and development of the fetus) and iron (for red blood cell RBC and deoxyribonucleic acid (DNA) synthesis). Consumption of foods associated with the “Western diet pattern” were however not a major constituent of the diet consumed among the people of the metropolis. Evidence from this study provides the basis for planning interventions that should prioritize dietary diversity and a priori determination of dietary patterns to improve maternal nutrition during pregnancy in order to prevent LBW. Results from such intervention studies could help determine the cause and effect of the observational relationships we have found and could help guide public health policy and direction regarding nutrition during pregnancy and LBW in Ghana.

Previous studies have only investigated the effect of single nutrients and or food group on LBW among this population. Our study provides evidence from a developing country context regarding the association of dietary pattern and DDS with LBW. The study however have some limitations. First, the use of a simple FFQ to estimate food intake may result in recall bias although we did probe and provided cues to aid mothers to recall. Second, DDS based on frequency responses may not represent true food intakes although DDS has been shown as a good indicator for measuring micronutrient quality (FAO & FHI, 2016) in socio-economically disadvantaged regions. Even though we provided several frequency responses to represent food intakes, we acknowledge that the use of frequency responses may not be exhaustive enough to describe the times these foods were eaten. It should however be noted that the measurement of usual intakes and quantity of food consumed provides a better assessment of nutritional status. Our inability to also estimate and adjust for total energy intake may influence our study results. Last, the lack of established cut-offs for additional percentage of variance explained in factor analysis for the determination of factors and which foods should be considered relevant in each pattern (Kjøllesdal and Ottesen, 2014) could have influenced the study result. It is also important to indicate that errors that could occur in the measurements of secondary data (data collected during ANC attendance and delivery) involved in this study could have influenced our results. These notwithstanding, the use of the FFQ as an efficient way of determining dietary diversity and dietary patterns have been validated (Clausen et al., 2005; Maruapula and Chapman, 2007). Our result is significant and similar to those conducted in other countries and might form the basis of exploring the relationship between DDS, dietary patterns and LBW in Ghana using experimental designs to determine the causality of these observed associations. It is also likely women who did not attend post-natal clinic at the facility were missed out. There is the need to consider these associations in larger multi-ethnic cohort given that our population were hospital-based and from a single hospital.

## 5 Conclusion

In this hospital based cross-sectional study in the Cape Coast metropolitan hospital, our findings revealed that mothers who had lower DDS during pregnancy had higher odds of delivering an infant with LBW. In addition, mothers who had “healthy” and “traditional” dietary patterns during pregnancy had lower odds of delivering an infant with LBW. These findings suggest that following a dietary pattern rich in fruits and vegetables as well as higher in diversity is essential during pregnancy. There is therefore the need to counsel and educate mothers on the most appropriate nutritional practices, food intakes and healthy behaviors during pregnancy to help prevent or reduce the prevalence of LBW in the study area.

## Declarations

### Author contribution statement

D.Y. Quansah: Conceived and designed the experiments; Analyzed and interpreted the data; Wrote the paper.

D. Boateng: Conceived and designed the experiments; Analyzed and interpreted the data.

### Funding statement

This research did not receive any specific grant from funding agencies in the public, commercial, or not-for-profit sectors.

### Competing interest statement

The authors declare no conflict of interest.

### Additional information

No additional information is available for this paper.

## References

[bib1] Abubakari A., Jahn A. (2016). Maternal dietary patterns and practices and birth weight in Northern Ghana. PloS One.

[bib2] Afriyie J., Bedu-Addo K., Asiamah E. (2016). Low birth weight among adolescents at Cape Coast metropolitan hospital of Ghana. Int J Reprod Contracept Obstet Gynecol.

[bib3] Ali F., Thaver I., Khan S.A. (2014). Assessment of dietary diversity and nutritional status of pregnant women in Islamabad, Pakistan. J. Ayub Med. Coll. Abbottabad.

[bib4] Amegah A.K., Klevor M.K., Wagner C.L. (2017). Maternal vitamin D insufficiency and risk of adverse pregnancy and birth outcomes: a systematic review and meta-analysis of longitudinal studies. PloS One.

[bib5] Annan R.A., Jackson A.A., Margetts B.M., Vorster H. (2015). Dietary patterns and nutrient intakes of a South African population and asymptomatic people infected with human immunodeficiency virus: the transition and urbanization in South Africa (THUSA) study. Afr. J. Food Nutr. Sci..

[bib6] Andersen L.T., Thilsted S.H., Nielsen B.B. (2003). Food and nutrient intakes among pregnant women in rural Tamil Nadu, South India. Publ. Health Nutr..

[bib8] Binkley N., Harke J., Krueger D. (2009). Vitamin K treatment reduces undercarboxylated osteocalcin but does not alter bone turnover, density, or geometry in healthy postmenopausal North American women. J. Bone Miner. Res..

[bib9] Chen X., Zhao D., Mao X., Xia Y., Baker P.N., Zhang H. (2016). Maternal dietary patterns and pregnancy outcome. Nutrients.

[bib10] Chen L.W., Aris I.M., Bernard J.Y. (2016). Associations of maternal dietary patterns during pregnancy with offspring adiposity from birth until 54 Months of age. Nutrients.

[bib11] Cespedes E.M., Hu F.B. (2015). Dietary patterns: from nutritional epidemiologic analysis to national guidelines. Am. J. Clin. Nutr..

[bib12] Christian P., Stewart C.P. (2010). Maternal micronutrient deficiency, fetal development, and the risk of chronic disease. J. Nutr..

[bib13] Clausen T., Charlton K.E., Gobotswang K.S., Holmboe-Ottesen G. (2005). Predictors of food variety and dietary diversity among older persons in Botswana. Nutrition.

[bib16] da Silva Lopes K., Ota E., Shakya P. (2017). Effects of nutrition interventions during pregnancy on low birth weight: an overview of systematic reviews. BMJ Glob Health.

[bib17] FAO & FHI 360 (2016). Minimum Dietary Diversity for Women: A Guide for Measurement.

[bib18] Farrell V.A., Harris M., Lohman T.G. (2009). Comparison between dietary assessment methods for determining associations between nutrient intakes and bone mineral density in postmenopausal women. J. Am. Diet Assoc..

[bib19] Fosu M.O., Munyakazi L., Nsowah-Nuamah N., N. N. (2013). Low birth weight and associated maternal factors in Ghana. J. Biol. Argric. Healthc..

[bib20] Fowles E.,R., Gabrielson M. (2005). First trimester predictors of diet and birth outcomes in low-income pregnant women. J. Community Health Nurs..

[bib22] Hu F.B. (2002). Dietary pattern analysis: a new direction in nutritional epidemiology. Curr. Opin. Lipidol..

[bib23] IBM Corp. Released (2013). IBM SPSS Statistics for Windows, Version 22.0.

[bib24] Jornayvaz F.R., Vollenweider P., Murielle B. (2016). Low birth weight leads to obesity, diabetes and increased leptin levels in Adults : the CoLaus study. Cardiovasc. Diabetol..

[bib25] Kant A. (2004). Dietary patterns and health outcomes. J. Am. Diet Assoc..

[bib27] Kjøllesdal M.K.R., Ottesen G.H. (2014). Dietary patterns and birth weight: a review. AIMS Publ. Health.

[bib29] Kolte D., Sharma R., Vali S. (2009). Correlates between micronutrient intake of pregnant women and birth weight of infants from central India. Internet J. Nutr. Wellness.

[bib30] Kopec G., Shekhawat P.S., Mhanna M.J. (2017). Prevalence of diabetes and obesity in association with prematurity and growth restriction, 285–95. Diabetes Metab Syndr Obes.

[bib31] Loy S.L., Mohamed H.J.B. (2013). Relative validity of dietary patterns during pregnancy assessed with a food frequency questionnaire. Int. J. Food Sci. Nutr..

[bib32] Maruapula S., Chapman-Novakofski K. (2007). Health and dietary patterns of the elderly in Botswana. J. Nutr. Educ. Behav..

[bib34] Prah J., Ameyaw E.O., Afoakwah R. (2016). Factors affecting birth weight in Cape Coast, Ghana. Int J Reprod Contracept Obstet Gynecol.

[bib35] Quansah D.Y., Boateng D., Kwantwi L.B., Owusu-Sekyere A., Amegah A.K. (2019). Dietary diversity score during pregnancy is associated with neonatal low apgar score: a hospital-based cross-sectional study. Int. J. Child Health Nutr..

[bib36] Rammohan A., Goli S., Singh D., Singh U. (2017). Maternal Dietary Diversity and Risk of Low Birth Weight: Empirical Findings from India. In Population Association of America.

[bib37] Ruel M.T. (2002). Is Dietary Diversity an Indicator of Food Security or Dietary Quality? A Review of Measurement Issues and Research Needs. FCND Discussion Paper No 140.

[bib38] Saaka M. (2012). Maternal dietary diversity and infant outcome of pregnant women in northern Ghana. Int. J. Child Health Nutr..

[bib42] Thompson J.M.D., Wall C., Becroft D. (2010). Maternal dietary patterns in pregnancy and the association with small for gestational age infants. Br. J. Nutr..

[bib43] Tian J.Y., Cheng Q., Song X.M. (2006). Birth weight and risk of type 2 diabetes, abdominal obesity and hypertension among Chinese adults. Eur. J. Endocrinol..

[bib45] Valero de Bernabéa J., Sorianob T., Albaladejoc R. (2004). Risk factors for low birth weight: a review. Eur. J. Obstet. Gynecol. Reprod. Biol..

[bib46] World Health Organization (2000). Obesity: Preventing and Managing the Global Epidemic. Report of a WHO Consultation. WHO Technical Report Series 894.

[bib47] World Health Organization (1992). International Statistical Classification of Diseases and Related Health Problems, Tenth Revision.

[bib48] World Health Organization Technical Consultation (2004). Towards the Development of a Strategy for Promoting Optimal Fetal Growth, Report of a Meeting (Draft).

[bib52] Yeneabat Tebikew, Adugna Haweni, Asmamaw Tarekegn, Wubetu Muluken, Admas Melaku, Hailu Getachew, Bedaso Asres, Amare Tadele (2019). Maternal dietary diversity and micronutrient adequacy during pregnancy and related factors in East Gojjam Zone, Northwest Ethiopia, 2016. BMC Pregnancy Childbirth.

[bib49] Zhang F., Tapera T.M., Gou J. (2018). Application of a new dietary pattern analysis method in nutritional epidemiology. BMC Med. Res. Methodol..

[bib50] Zerfu T.A., Umeta M., Baye K. (2016). Dietary diversity during pregnancy is associated with reduced risk of maternal anemia, preterm delivery, and low birth weight in a prospective cohort study, in rural Ethiopia. Am. J. Clin. Nutr..

